# FireFace: Leveraging Internal Function Features for Configuration of Functions on Serverless Edge Platforms

**DOI:** 10.3390/s23187829

**Published:** 2023-09-12

**Authors:** Ming Li, Jianshan Zhang, Jingfeng Lin, Zheyi Chen, Xianghan Zheng

**Affiliations:** 1 College of Computer and Data Science, Fuzhou University, Fuzhou 350116, China; 210310011@fzu.edu.cn (M.L.); 221027069@fzu.edu.cn (J.L.); xianghan.zheng@fzu.edu.cn (X.Z.); 2 Key Laboratory of Spatial Data Mining and Information Sharing, Ministry of Education, Fuzhou 350002, China; 3 Fujian Key Laboratory of Network Computing and Intelligent Information Processing, Fuzhou University, Fuzhou 350116, China; 4 College of Computer and Control Engineering, Minjiang University, Fuzhou 350116, China; jszhang@mju.edu.cn

**Keywords:** serverless computing, function as a service, configuration optimization, SLO

## Abstract

The emerging serverless computing has become a captivating paradigm for deploying cloud applications, alleviating developers’ concerns about infrastructure resource management by configuring necessary parameters such as latency and memory constraints. Existing resource configuration solutions for cloud-based serverless applications can be broadly classified into modeling based on historical data or a combination of sparse measurements and interpolation/modeling. In pursuit of service response and conserving network bandwidth, platforms have progressively expanded from the traditional cloud to the edge. Compared to cloud platforms, serverless edge platforms often lead to more running overhead due to their limited resources, resulting in undesirable financial costs for developers when using the existing solutions. Meanwhile, it is extremely challenging to handle the heterogeneity of edge platforms, characterized by distinct pricing owing to their varying resource preferences. To tackle these challenges, we propose an adaptive and efficient approach called FireFace, consisting of prediction and decision modules. The prediction module extracts the internal features of all functions within the serverless application and uses this information to predict the execution time of the functions under specific configuration schemes. Based on the prediction module, the decision module analyzes the environment information and uses the Adaptive Particle Swarm Optimization algorithm and Genetic Algorithm Operator (APSO-GA) algorithm to select the most suitable configuration plan for each function, including CPU, memory, and edge platforms. In this way, it is possible to effectively minimize the financial overhead while fulfilling the Service Level Objectives (SLOs). Extensive experimental results show that our prediction model obtains optimal results under all three metrics, and the prediction error rate for real-world serverless applications is in the range of 4.25∼9.51%. Our approach can find the optimal resource configuration scheme for each application, which saves 7.2∼44.8% on average compared to other classic algorithms. Moreover, FireFace exhibits rapid adaptability, efficiently adjusting resource allocation schemes in response to dynamic environments.

## 1. Introduction

With the progression of cloud computing, back-end infrastructure maintenance is increasingly decoupled from application development. As an emerging application deployment architecture, serverless computing ( function-as-a-service, FaaS) has garnered widespread attention in recent years owing to its nimble and uncluttered management. Prominent public cloud platforms offer various serverless computing products, such as AWS Lambda [1], Azure Functions [2], and Google Cloud Functions [3]. Following this paradigm, developers are liberated from concerns surrounding infrastructure resource management, as the platform shields them from such intricacies. Hence, developers can focus on their cloud functions by employing a high-level programming language (e.g., Java or Python), configuring necessary parameters, and uploading these functions onto the serverless platform [4]. When deployed, these functions can be invoked by APIs or HTTP requests [5]. Furthermore, developers can establish invocation relationships between these deployed serverless functions to facilitate seamless collaboration and data exchange, culminating in a fully-fledged serverless application. As serverless computing continues its ascent, platform providers have recently redirected their attention from the cloud to the edge [6], which offers less transmission latency and as such aligns harmoniously with the lightweight and rapid response ethos underpinning serverless computing.

Although serverless computing alleviates developers’ concerns about the intricate details of the back-end infrastructure, it requires the configuration of essential parameters such as latency and memory constraints that directly influence the deployment cost of functions [7,8]. However, due to the black box nature of the serverless platform, it is extremely challenging for developers to determine the optimal configuration of each function within an application. A recent survey indicated that a staggering 47% of serverless functions in production adopt default memory sizes [9], indicating that developers often neglect resource sizing, resulting in avoidable extra costs.

The existing solutions for configuring resources in serverless applications can be classified into two principal categories. The first category entails modeling performance based on historical datasets of functions, which can be used to predict the execution time of functions under diverse configurations, thereby facilitating the selection of an appropriate configuration plan [9,10,11]. However, this category of solutions is limited due to its reliance on the availability of extensive historical data, and fails to address data scarcity when new functions are submitted. The second category involves determining the optimal memory size for a serverless function through a combination of sparse measurements and interpolation/modeling techniques. This category of solutions first executes functions with the given sampling point configurations selected through traversal [12], Bayesian optimization [13], TPE [14], etc. Next, it aggregates execution data and constructs a performance model to identify an appropriate resource configuration scheme. Nevertheless, its applicability to serverless edge platforms is hindered by exorbitant pricing compared to cloud platforms due to limited resource availability. Furthermore, the existing solutions must pay more attention to the disparity in resource preferences across distinct edge platforms, resulting in divergent resource pricing.

To address these challenges, in this paper we design and implement a resource configuration approach for serverless applications called FireFace. FireFace serves as a guiding beacon for developers, aiding them in selecting an optimal resource configuration scheme (including CPU, memory, and edge platform) for each function within the application. By leveraging this approach, we harmonize the dual objectives of cost minimization and satisfaction to SLO. Unlike other approaches or platforms that intertwine memory with CPU and increase CPU by tuning memory, we advocate for a clear separation between CPU and memory decisions. This pattern can avoid resource wastage, especially when confronted with computation-intensive functions. FireFace comprises two core modules, a prediction module and a decision module. For a newly submitted serverless application, the prediction module extracts the internal features of all functions and then harnesses this information to predict the execution time under specific configuration schemes using well-trained models. Based on the prediction module, the decision module analyzes the environmental information, for which it employs the Adaptive Particle Swarm Optimization algorithm using Genetic Algorithm operators (APSO-GA) to generate the optimal configuration scheme for each function within an application.

In summary, the key contributions of our work are as follows:

(1) We provide a framework for predicting the execution time of newly submitted serverless functions. By analyzing internal feature information extracted through static code analysis, our framework exhibits the excellent ability to predict function execution time for given configurations (Section 3.3).

(2) We propose a cost-effective resource configuration algorithm based on APSO-GA. This algorithm empowers developers to configure optimal schemes for each function, including CPU, memory, and platform, thereby minimizing costs while fulfilling SLO. Moreover, the algorithm demonstrates adaptability when the edge environment changes (Section 3.4).

(3) We implement the FireFace prototype. Our experimental results show that FireFace can provide accurate predictions for serverless applications, with a prediction error of around 5%. Meanwhile, the resource configuration scheme generated by FireFace can save 7.2∼44.8% on overhead compared to other classic algorithms (Section 4).

The rest of this paper is organized as follows. Section 2 introduces related work. Section 3 presents the detailed design of FireFace. Section 4 evaluates FireFace. Finally, Section 5 concludes the paper.

## 2. Related Work

Configuring appropriate resources for functions is a worthwhile research topic within the serverless computing domain [15] involving CPU and memory size configuration, deployment platform selection, etc. Costless [16] analyzes the factors that affect the price of serverless computing services, proposing a systematic approach to reduce the cost of serverless workflows. This approach effectively assesses the feasibility of fusing multiple functions into a larger function, and decides on memory limit assignment for each serverless function. Haneul et al. [17] formulated a Constrained Markov Decision Process (CMDP) problem and converted it into a Linear Programming (LP) model to obtain an optimal stochastic policy. The proposed method strives to minimize memory resource consumption and ensure timely task completion by strategically managing container instances. AMPS-Inf [18] leverages a constrained optimization formulation to explore model partitioning and memory configuration for neural networks deployed on serverless platforms with the objective of cost reduction while satisfying SLOs. However, these approaches suppose that function execution time under various configurations are known, a condition that may not be applicable in real-world conditions.

Several approaches have been explored using historical data to train prediction models for determining optimal function configurations. AWS Compute Optimizer [19] utilizes machine learning techniques to analyze historical utilization metrics and recommend the best resource configuration for applications, thereby enhancing cost efficiency and performance. It can identify the optimal memory configuration for lambda-based functions as well. However, a limitation of this approach is that it only applies to functions invoked on the AWS platform at least 50 times in the past two weeks. Sizeless [9] builds a multi-objective regression model that captures the runtime information of a serverless function under a specific memory configuration as input. The model then predicts the execution time of this function in other memory configurations, aiming to select the optimal configuration that minimizes cost while satisfying latency constraints. SAAF [20] is a tool designed to analyze the performance of FaaS workloads, resource utilization, and infrastructure as a means of facilitating accurate performance predictions. It estimates the execution time of FaaS functions through Linux CPU time accounting principles and multiple regression. This tool is further employed by in the same authors’ follow-up work [11] to identify appropriate memory configurations, leading to a reduction both in execution time and cost. λDNN [21] is a cost-efficient function resource provisioning framework that selects a suitable resource configuration (i.e., function number and memory size) for serverless functions by providing predictable performance for serverless Distributed Deep Neural Network (DDNN) training workloads. It can save on the budget of provisioned functions while guaranteeing DDNN training performance with serverless functions. DiSDeL [22] is a runtime framework tailored for deep learning tasks. It allocates memory based on the DL model, dataset, runtime, and execution logs stored in the container. StepConf [10] establishes a memory–execution time relationship,= by considering scenarios with multicore-friendly programs and those limited to single-threaded performance. By fitting exponential and inverse functions, appropriate memory and concurrency numbers can be determined to improve the performance of DL workloads on a serverless platform. However, these methods necessitate the collection of a stable amount of training data for each serverless function, and are usually simple models designed for a specific type of function.

To address the aforementioned challenges, various alternative approaches have been proposed seeking to determine the optimal memory size for serverless functions through the combination of sparse measurements and interpolation/modeling. COSE [23], as one of the earliest such methods, employs Bayesian techniques for resource allocation in serverless applications. It effectively learns a performance model that describes the relationship between memory size and execution time using limited measurement points. Consequently, COSE can decide the appropriate configuration and placement for serverless applications. Ali et al. [13] extended the capabilities of COSE to accommodate applications with arbitrary acyclic service graphs. The AWS Lambda Power Tuning tool [24] adopts an exhaustive search algorithm to ascertain the optimal memory level based on cost or execution time considerations. However, this process mandates a minimum of 225 requests to the function by default. Maff [12] is a Python-based framework that automates the process of searching for the optimal memory configuration of a FaaS function. The framework employs three optimization algorithms, including linear, binary, and gradient descent, to find the minimum cost. FaaSDeliver [14] considers the heterogeneity of serverless platforms and makes separate decisions for memory and CPU. It selects the most cost-efficient function delivery policy for different functions and computing devices based on the Tree-structured Parzen Estimator (TPE). However, it is essential to note that these approaches necessitate actual application execution to measure performance at different memory sizes. Experiments with FaaSDeliver have demonstrated that it typically requires several dozen iterations at least, with more complex cases requiring 100 to 200 iterations. While this may be practical for cloud platforms, this many iterations can incur significant overhead on resource-constrained edge platforms. Moreover, the separation of CPU and memory makes the original one-dimensional problem into a more challenging two-dimensional problem, complicating interpolation.

The solution that we propose in this paper has several highlights compared to the existing methods:

(1) It eliminates the need to use previously collected historical datasets in newly submitted serverless applications.

(2) The process of generating an optimized configurations avoids significant costs, as the actual execution of the application is not necessary.

(3) The variability and dynamics of edge platform pricing are taken into consideration.

## 3. Approach

### 3.1. Problem Formulation

In this paper, we translate the task of resource configuration for serverless applications into an optimization problem centered around cost efficiency. There are *m* edge platforms provided for developers, symbolized as $E=\{e_{1},e_{2},\cdots ,e_{m}\}$, with each platform represented by the tuple $e_{i}=\left(cpu,mem\right)$, where $e_{i}.cpu$ and $e_{i}.mem$ denote the pricing of the platform’s CPU and memory, respectively. The pricing for CPU and memory varies due to the distinctive resource preferences exhibited by each edge platform. The serverless application is denoted by the symbol $F=\{f_{1},f_{2},\cdots ,f_{n}\}$, where $f_{i}$ denotes the *i*-th function, and $p_{i}=\left(cpu,memory,plat\right)$ indicates the configuration scheme assigned to function $f_{i}$. Referring to the settings of serverless platforms such as AWS and Google, we set the value range for CPU to be 0.2 to one core in increments of 0.2, while the value range for memory spans from 128 MB to 1024 MB, progressing in 128 MB increments. For a function $f_{i}$, the cost of executing one time is calculated as follows:

(1) $$c_{i}\left(p_{i}\right)=t_{i}\left(p_{i}\right)*msc\left(p_{i}\right)$$

(2) $$msc\left(p_{i}\right)=p_{i}.cpu*e_{p_{i}.plat}.cpu+p_{i}.memory*e_{p_{i}.plat}.mem$$

where $t_{i}\left(p_{i}\right)$ denotes the function execution time under the configuration $p_{i}$ and $msc(p_{i})$ denotes the cost per millisecond incurred by the configuration, encompassing both CPU and memory usage. As an example, we consider an edge platform $e_{1}$ with a CPU pricing of 0.02 per core-millisecond and a memory pricing of 0.0008 per MB-millisecond. Consequently, when $f_{i}$ is configured as $p_{i}=\left(0.4,256,1\right)$ and the function executes for one millisecond under this configuration, it incurs a cost of 1 × (0.4 × 0.02 + 256 × 0.0008) = 0.2848.

Finally, we formulate the problem as follows:

(3) $$minimize\sum_{i=1}^{n}c_{i}\left(p_{i}\right)*calls_{i}$$

(4) $$subject\sum_{i=1}^{n}t_{i}\left(p_{i}\right)*calls_{i}\leq SLO$$

where *SLO* represents the upper boundary of acceptable latency of applications specified by the developer and $calls_{i}$ denotes the number of times the function $f_{i}$ has been called. As shown in Equation (4), the application’s execution time amounts to the summation of the execution time of its constituent functions, with each multiplied by the corresponding number of calls. In this study, we neglect the data transfer latency between functions in light of the rapid data transfer rate between edge platforms. Our objective is to minimize the overall cost while satisfying the SLO. Although this problem can be solved through traversal, the reality is that such an approach soon becomes prohibitively expensive. For instance, when a four-function application comprises five, eight, and five choices of CPUs, memories, and platforms, respectively, the number of feasible configurations amounts to $160^{4}=655,360,000$. Moreover, executing the application under each configuration scheme for verification purposes imposes significant overhead. To address this challenge in a cost-effective way, we propose FireFace, a method that leverages the internal features of each function within a serviceless application to identify the most optimal resource configuration scheme.

### 3.2. FireFace Overview

**Figure 1:**
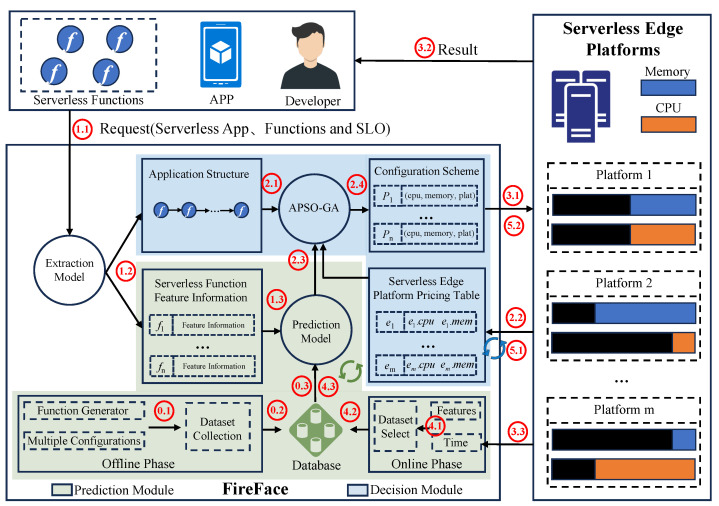
Overview of the FireFace framework.

The overall architecture of FireFace is presented in Figure 1. FireFace is designed to guide developers to configure functions within serverless applications, which can be deployed on edge platforms to minimize the cost while satisfying the SLO. FireFace consists of two main components: first, the **prediction module** is responsible for extracting features of each function within the serverless application; drawing upon this information to efficiently predict the function execution time under a specific configuration; second, the **decision module**, based on an APSO-GA algorithm, converges iteratively to obtain the “optimal” configuration scheme. The decisionmaking process draws insights from the application structure, edge platform pricing table, and prediction model. Furthermore, Steps 4 and 5 are designed to enhance the adaptability of the prediction model and configuration scheme in response to the dynamic nature of the edge environment. The process is as follows:

**Step 0: Prediction model training (offline).** Prior to application, the prediction model requires pretraining. Our generalized model is not tied to specific functions, and can be trained using historical data from prior execution logs, publicly available datasets, or well-trained models from external sources. This training process is devoid of any additional cost for developers. To quickly build the prediction model, we design a function generator and a set of configurations (step 0.1). Stable data under these configurations are then collected (step 0.2), forming the dataset used for model training (step 0.3).

**Step 1: Extracting feature information.** The developer submits a serverless application, the corresponding constituent functions and the SLO (step 1.1). Then, the calling relationships between functions and their feature information are extracted using our previous work FunOff [25] and DNNOff [26] (step 1.2), serving as an input to the prediction model for execution time estimation under given configurations (step 1.3).

**Step 2: Configuration scheme decision (static).** After obtaining the application structure (step 2.1), edge platform pricing table (step 2.2), and function execution time (step 2.3), the APSO-GA algorithm efficiently determines the optimal configuration scheme (step 2.4).

**Step 3: Function deployment and execution.** Based on the configuration scheme from Step 2, each function is deployed to the corresponding platform with specific CPU and memory settings (step 3.1). When the serverless application executes, the platform runs the functions sequentially and returns the application’s execution result to the user (step 3.2). Simultaneously, FireFace captures the execution time of each function for subsequent prediction model optimization (step 3.3).

**Step 4: Prediction model optimization (online).** The prediction module amalgamates the collected function execution time with previously extracted function features to generate new data (step 4.1). When a substantial volume of stable data has been amassed, these datasets are added to the database (Step 4.2). Subsequently, an incremental learning approach is employed (Step 4.3) to enhance and fine-tune the prediction model.

**Step 5: Configuration scheme optimization (dynamic).** The ebb and flow of edge platform resource utilization introduces a dynamic facet that encompasses pricing adjustments aligned with resource preferences (step 5.1). To cope with this, our algorithm swiftly adapts to the new environment, combining insights gleaned from the changed pricing table and the previous configuration scheme (step 5.2).

### 3.3. Prediction Module

One of the challenge involved in configuring appropriate resources for a newly submitted application is the absence of historical data. In response to this predicament, the prediction module acquires the application structure and internal features of each function through static code analysis. Using the pretrained prediction model, this module can estimate the application’s execution time under a specified configuration scheme without the need for actual execution on the serverless edge platform.

#### 3.3.1. Extraction of Application Structure and Internal Features

This section introduces the extraction of the application structure and the functions feature, leveraging the methodologies proposed in previous works [25,26]; in this paper, we mainly focus on Java and DNN-based applications.

**Application structure:** our prior work obtains the application structure through the Soot tool [27] or through analysis of configuration files. In Java applications, the structure manifests as a directed acyclic graph in which each node represents an object method and the edges signify the invocation relationships and the number of calls between methods. For DNN applications, the structure assumes the form of a workflow, with nodes representing submodules comprising multilayer DNN layers and edges indicating the direction of the data flow. In serverless applications the structure tends to be simpler, and is often represented as a chain of functions, which is a more streamlined version of directed graphs or workflows. Thus, the methodologies proposed in our prior works can be seamlessly extended to address this simpler form. In the present paper, the application structure is extracted in order to count the number of function invocations required by Equations (3) and (4).

**Function internal features:** the internal features of a function encapsulate vital information about its complexity. In our previous experimental results we were able to identify certain features that have a substantial impact on function execution time, which are summarized in Table 1.

The principal parameters that influence the execution time of a Java function (method) encompass the following: (1) blockDepth denotes the function’s depth; (2) percentBranchStatements indicates the percentage of branch statements; (3) complexity signifies the cyclomatic complexity of the method; (4) statement represents the number of statements in the method; and (5) calls indicates the number of internal calls within the method.

For the DNN function (submodule), its composition comprises diverse DNN layers. Because the parameters and execution time of different types of DNN layers are distinct, we developed the various types of layers using separate models. Table 1 is missing several types of layers, such as flatten layers and dropout layers. This is because empirical experimentation has revealed that the time incurred by these layers is typically at the microsecond level, and as such can be neglected.

Notably, this subsection primarily constitutes an application of preliminary works that warrant a succinct portrayal herein. Further technical details can be found in [25,26].

#### 3.3.2. Prediction Model Construction (Offline)

**(1) Generation of the Training Dataset**

To ensure the robust training of our prediction model, we built a comprehensive dataset encompassing diverse function types and their execution under varied resource configurations. Specifically, for Java functions we implemented sixteen common computation-intensive functions, including string hashing, floating-point arithmetic, and recursive calculations. These functions are susceptible to both CPU and memory size. For DNN models, we crafted a coding program that randomly generates multi-layer DNN modules. Although the untrained DNN models bear no intrinsic significance, they can provide rich training data for our prediction model.

**Figure 2:**
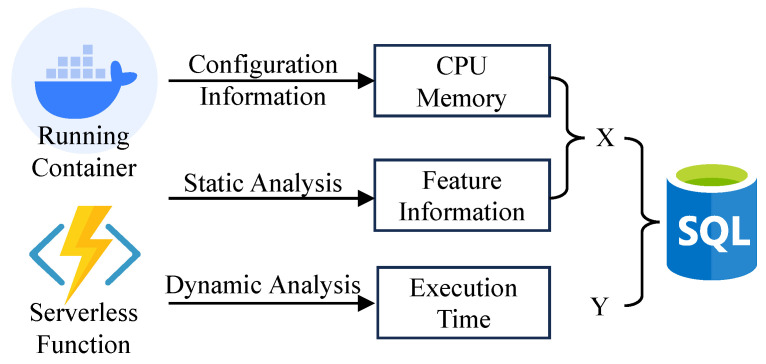
Generation of the dataset.

We executed these functions twenty times with various memory and CPU combinations, taking the average value as the final execution time. Figure 2 shows the function execution time recorded for each configuration and integrated with the internal features to generate a rich historical dataset. Next, we describe the process of training the prediction model using this dataset.

**(2) Random Forest Regression Algorithm**

The Random Forest Regression (RFR) Algorithm [28] is a prominent machine learning paradigm that has proven to be both effective and flexible in characterizing nonlinear relationships. Here, we deploy this algorithm to construct the prediction model by leveraging the dataset previously generated in the preceding subsection. We utilized 80% of the dataset for training the prediction model while reserving the remaining 20% to assess the accuracy of the trained model.

To improve the performance of the model, we optimized five key parameters of the Random Forest algorithm:

- n_estimators: denotes the number of decision trees; increasing this value bolsters the stability and accuracy of the model.
- max_features: denotes the number of features considered in each decision tree; this parameter offers control over the degree of overfitting and underfitting.
- max_depth: denotes the maximum depth of each decision tree; setting an appropriate maximum depth enhances the model’s generalization capability.
- min_split: denotes the minimum number of samples required for internal node splitting; raising this value mitigates over-splitting, thereby augmenting the model’s generalization capacity.
- min_leaf: denotes the minimum number of samples mandated for leaf nodes; appropriate tuning of this parameter prevents overfitting and guarantees that each leaf node possesses a sufficient number of samples for reliable prediction.

Considering these parameters’ effects on accuracy and prediction time, our prediction models are defined as shown in Table 2. In Section 4, we further discuss the prediction accuracy of these models under their respective parameter configurations and compare their performance with other regression methods.

#### 3.3.3. Prediction Model Optimization (Online)

To cope with real-time data streams and dynamic environments, we have embraced an incremental learning approach [29] to ensure the ability of the prediction model to adapt to environmental changes. The specific steps are as follows.

**Generation of New Data.** As FireFace collects the execution time of a function from the serverless platform, it combines this information with the pertinent function features to generate a new piece of data. To reduce the impact of execution time jitter on model training, this new data item is not directly incorporated into the training dataset. Instead, it is temporarily stored in an alternate dataset. Upon amassing a sufficient volume of data, we curate the dataset by excluding extreme values and calculating the average of the remaining data, thereby obtaining the final data entry for the function.

**Model Optimization.** When the processed new data reach a predetermined threshold, they are added to the database and incremental learning of the Random Forest model is performed. Incremental learning is a localized update mechanism that adapts the model to new data without retraining the whole model. The optimization process is as follows: first, the entire dataset is divided into small batches of data blocks to expedite the model optimization process; second, each data block is employed as an input to each decision tree within the random forest for training and updating of the model parameters. The decision tree model calculates the gradient based on the loss function and updates the model parameters based on the learning rate setting. In this way, the model gradually refines itself to suit the evolving environment. Considering the independent nature of each decision tree’s training process, we leverage parallel processing to handle each data block, thereby improving the overall training efficiency. Finally, the incremental learning process for the entire dataset concludes when all data blocks have been traversed, and the updated model is able to serve the decision module more accurately.

### 3.4. Decision Module

The decision module is designed to generate a configuration scheme for each function. The decision module determines the optimal configuration scheme by leveraging the application structure, the edge platform pricing table, and the function execution times, seeking to minimise the cost while satisfying the SLO. Moreover, the algorithm can adaptively update the configuration scheme in response to changes in the edge environment, resulting in improved accuracy.

#### 3.4.1. Configuration Scheme Based on APSO-GA

The search for the optimal configuration scheme for functions has been proven to be an NP-hard problem [14]. Although traditional Particle Swarm Optimization (PSO) methods have been extensively applied to tackle continuous optimization problems [30], the decision variables are discrete in the problem studied in this paper. A novel coding method is needed to make PSO applicable to this problem. Furthermore, a well-suited particle update strategy must be introduced in order to circumvent the premature convergence issue of the traditional PSO. To address the above inadequacies of the traditional PSO, we propose the APSO-GA algorithm to explore the optimal resource configuration scheme for applications in the edge serverless environment, described as follows.

**(1) Problem Encoding**

We employ particle Z to denote the candidate configuration schemes for functions within in serverless application in the edge environment; the *i*-th particle at the *t*-th iteration $Z_{i}^{t}$ is described by Equation (5), where *n* is the total number of functions in the application.

(5) $$Z_{i}^{t}=\left(z_{i,1}^{t},z_{i,2}^{t},\cdots ,z_{i,n}^{t}\right)$$

(6) $$z_{i,k}^{t}=\left(cpu,mem,plat\right)$$

**Figure 3:**
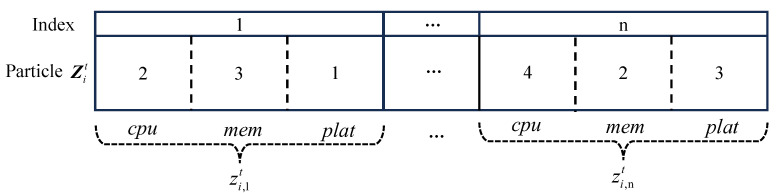
Example of the particle used for the resource configuration scheme.

In Equation (6), $z_{i,k}^{t}\left(k=1,2,\cdots ,n\right)$ represents the k-th gene in the i-th particle at the t-th iteration containing the CPU, memory, and platform configurations for the function $f_{k}$. Figure 3 illustrates an example of particle $z_{i,k}^{t}$, where $z_{i,1}^{t}$ represents the resource configuration of function $f_{1}$ under this scheme. Specifically, function $f_{1}$ is deployed on $e_{1}$ and configured with a resource size of 0.1 × 2 = 0.2 cores of CPU and 6 × 43 = 192 MB of memory.

It is worth noting here that certain resource configuration schemes corresponding to particles may not satisfy the SLO, leading to the execution time of the application exceeding the latency limit. For clarity, in this paper, those particles with applications that comply with the delay limit under the corresponding policy are deemed feasible particles, while those that exceed the delay limit are referred to as infeasible particles.

**(2) Fitness Function**

To facilitate a comparative assessment of different particles, it is imperative to evaluate them using a fitness function. Typically, particles with smaller fitness function values indicate more favourable candidate schemes. In this paper, we aim to obtain a configuration scheme for a serverless application that satisfies the SLO while minimizing the overall cost. Consequently, a particle boasting a lower deployment cost is regarded as a more favourable scheme. Considering the presence of infeasible particles, we categorize the comparison of particles into the following three cases.

Case 1: one particle represents a feasible solution and the other is infeasible. In this scenario, the feasible particle is selected; the fitness function is defined below.

(7) $$F\left(Z_{i}^{t}\right)=\left\{\begin{array}{c} 0,\;if\sum_{k=1}^{n}t_{k}\left(z_{i,k}^{t}\right)*calls_{k}\leq SLO\\ 1,\;else \end{array}\right.$$

Case 2: both particles are deemed infeasible. Here, the particle closer to the latency limit is preferred, as it is more likely to evolve into a feasible scheme in subsequent iterations. The fitness function is defined as follows:

(8) $$F\left(Z_{i}^{t}\right)=\sum_{k=1}^{n}t_{k}\left(z_{i,k}^{t}\right)*calls_{k}-SLO.$$

Case 3: both particles are feasible. In this case, the particle with the lower monetary cost is chosen, and the fitness function is as follows:

(9) $$F\left(Z_{i}^{t}\right)=\sum_{k=1}^{n}c_{k}\left(z_{i,k}^{t}\right)*calls_{k}.$$

**(3) Update Strategy**

Traditional PSO comprises three essential components: inertia, individual cognition, and social cognition [31]. The iterative updating of each particle is influenced by its personal best particle and the global best particle of the current generation. However, a significant limitation of PSO lies in its early convergence to local optima. To enhance the algorithm’s search capability, we introduce a Genetic Algorithm (GA) mutation operator and crossover operator for particle updating. The iterative update of the *i*-th particle in the (*t* + 1)-th iteration is provided by

(10) $$Z_{i}^{t+1}=F_{gc}\left(F_{pc}\left(F_{mu}\left(Z_{i}^{t},\delta^{t+1},iNum_{mu}\right),{\mathrm{pBest}}_{i}^{t},\eta_{pc}^{t+1},iNum_{pc}\right),{\mathrm{gBest}}^{t},\eta_{gc}^{t+1},iNum_{gc}\right),$$

where $F_{mu}$ represents the mutation operation, $F_{gc}$ and $F_{pc}$ denote the crossover operation, $\delta^{t+1}$ signifies the inertia weight, and $\eta_{pc}^{t+1}$ and $\eta_{gc}^{t+1}$ are the acceleration coefficients.

**A. Mutation**

**Figure 4:**
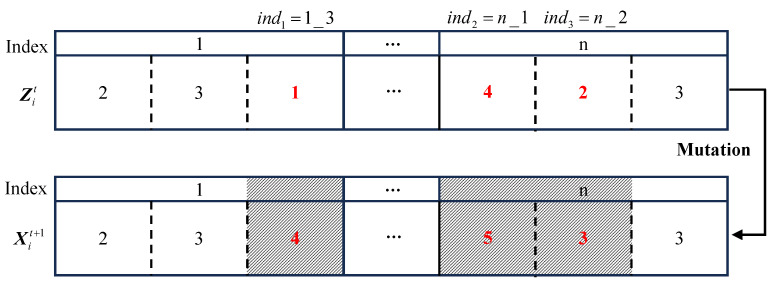
Example mutation operation. The shaded portions of Figure 4 and Figure 5 are used to differentiate from unshaded particles. The presence or absence of a shadow indicates two different particles.

The GA mutation operator is introduced into the inertia update operation of PSO. The outcome of this operation is defined as follows:

(11) $$X_{i}^{t+1}=F_{mu}\left(Z_{i}^{t},\delta^{t+1},iNum_{mu}\right)=\left\{\begin{array}{c} Mu\left(Z_{i}^{t},iNum_{mu}\right),\;\phi_{mu}⩽\delta^{t+1}\\ Z_{i}^{t},\;\;\;\;else \end{array}\right.$$

where $X_{i}^{t+1}$ denotes the new particle achieved after the mutation operation, Mu() denotes the mutation operator, $\delta^{t+1}$ denotes the threshold that triggers mutation, $iNum_{mu}$ denotes the number of positions subject to mutation, and $\phi_{u}$ is a random number from 0 to 1. If the $\phi_{mu}$ is less than $\delta^{t+1}$, the mutation operation is activated. Initially, $iNum_{mu}$ positions are randomly selected, including the index of the particle and the position within the particle. Subsequently, depending on whether it pertains to CPU, memory, or platform, a corresponding random operation is performed to replace the original decision. Figure 4 shows an example of the mutation operator; in the example, $iNum_{mu}$ is 3 and the three positions selected are 1_3, n_1, and n_2. The mutation operation leads to the following changes in the resource configuration scheme: the deployment platform of function $f_{1}$ shifts from 1 to 4; the CPU size of function $f_{n}$ changes from 0.1 × 4 = 0.4 cores to 0.1 × 5 = 0.5 cores; and the memory size changes from 64 × 2 = 128 MB to 64 × 3 = 192 MB.

**B. Crossover**

**Figure 5:**
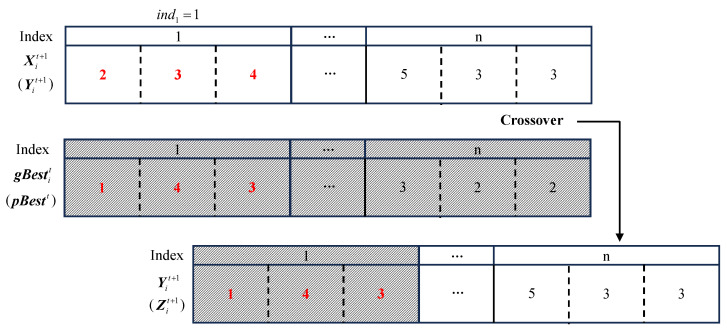
Example crossover operation.

The GA crossover operator is introduced into the personal cognitive update and the social cognitive update, which are represented as follows:

(12) $$Y_{i}^{t+1}=F_{pc}\left(X_{i}^{t+1},{\mathrm{pBest}}_{i}^{t},\eta_{pc}^{t+1},iNum_{pc}\right)=\left\{\begin{array}{c} Cr\left(X_{i}^{t+1},{\mathrm{pBest}}_{i}^{t},iNum_{pc}\right),\;\phi_{pc}⩽\eta_{pc}^{t+1}\\ X_{i}^{t+1},\;\;\;\;\;\;else \end{array}\right.$$

(13) $$Z_{i}^{t+1}=F_{gc}\left(Y_{i}^{t+1},{\mathrm{gBest}}^{t},\eta_{gc}^{t+1},iNum_{gc}\right)=\left\{\begin{array}{c} Cr\left(Y_{i}^{t+1},{\mathrm{gBest}}^{t},iNum_{gc}\right),\;\phi_{gc}⩽\eta_{gc}^{t+1}\\ Y_{i}^{t+1},\;\;\;\;\;\;else \end{array}\right.$$

where $Y_{i}^{t+1}$ and $Z_{i}^{t+1}$ respectively signify the new particles after the personal and social cognitive update; it is important to note that the new particle $Y_{i}^{t+1}$ is evolved from $X_{i}^{t+1}$, while the new particle $Z_{i}^{t+1}$ is evolved from $Y_{i}^{t+1}$, which is the final result of the (t+1)-th iteration. Furthermore, Cr() denotes the crossover operation, ${\mathrm{pBest}}_{i}^{t}$ denotes the optimal historical particle of the i-th individual in the t-th iteration, ${\mathrm{gBest}}^{t}$ denotes the overall optimal among all particles, $\eta_{pc}^{t+1}$ ($\eta_{gc}^{t+1}$) represents the threshold for individual (social) crossover, and $iNum_{pc}$ ($iNum_{gc}$) represents the number of genes involved in an individual (social) crossover. Unlike the mutation operation, the crossover operation pertains to the entire genes, as there is linkage between CPU, memory, and platform. Superior particles indicate reasonable matching among these three aspects; thus, we consider their overall alteration. The crossover operation is executed when the random value $\phi_{pc}$ ($\phi_{gc}$) is smaller than the threshold $\eta_{pc}^{t+1}$ ($\eta_{gc}^{t+1}$). Figure 5 illustrates an example of the crossover operation in which the number of genes subject to crossover is 1 and the gene index is 1, signifying that the configuration scheme for the function $f_{1}$ in the old particle is replaced by the one in ${\mathrm{pBest}}_{i}^{t}$ (${\mathrm{gBest}}^{t}$).

**(4) Mapping from a Particle to a Resource Configuration Scheme**

The mapping of a particle to a specific resource configuration scheme is detailed in Algorithm 1. For a given particle $Z_{i}^{t}$ as input, the corresponding scheme P is obtained as output. For each gene $z_{i,k}^{t}$ in the particle, the following operations are performed: in line 2, the CPU information $z_{i,k}^{t}.cpu$ recorded in the gene is multiplied by 0.1 to represent the number of cores $p_{k}.cpu$ allocated to the function $f_{k}$; in line 3, the memory information $z_{i,k}^{t}.mem$ recorded in the gene is multiplied by 64 to represent the memory size $p_{k}.memory$ assigned to the function $f_{k}$; in line 4, the deployment platform information $z_{i,k}^{t}.plat$ recorded in the gene is directly assigned to the deployment platform $p_{k}.plat$ of function $f_{k}$; and upon completion of these operations for all genes, the specific resource configuration scheme P is obtained.
**Algorithm 1** Mapping of a particle to a resource configuration scheme.**Input:** Paticle $Z_{i}^{t}=\left\{z_{i,1}^{t},z_{i,2}^{t},\cdots ,z_{i,n}^{t}\right\}$.**Output:** Resource configuration scheme $P=\{p_{1},p_{2},\cdots ,p_{n}\}$.1:**for** each $z_{i,k}^{t}\in Z_{i}^{t}$ **do**2:    $p_{k}.cpu\leftarrow z_{i,k}^{t}.cpu*0.1$3:    $p_{k}.memory\leftarrow z_{i,k}^{t}.mem*64$4:    $p_{k}.palt\leftarrow z_{i,k}^{t}.palt$5:**end for**

**(5) Parameter Setting**

The inertia weight δ significantly impacts the searchability and convergence of the PSO algorithm [32]. A larger value enhances global search ability, while a smaller value strengthens local search ability. The classical inertia weight adjustment method is as follows:

(14) $$\delta =\delta_{max}-iters_{cur}\frac{\delta_{max}-\varepsilon_{min}}{iters_{max}}$$

where $\delta_{max}$ and $\delta_{min}$ denote the maximum and minimum values of the initial setting of δ, respectively, and $iters_{cur}$ and $iters_{max}$ denote the current and maximum number of iterations, respectively.

The conventional update strategy for δ is solely related to the number of iterations, which is not optimally adapted to the nonlinear nature of minimum cost among functions. Therefore, we propose a discrete adjustment method based on the merits of the current population particles and adaptive adjustment, as follows:

(15) $$\delta^{t+1}=\delta_{max}-\left(\delta_{max}-\delta_{min}\right)\exp\left(\frac{d(Z_{i}^{t})}{d(Z_{i}^{t})-1.01}\right)$$

(16) $$d\left(Z_{i}^{t}\right)=\frac{div(Z_{i}^{t},{\mathrm{gBest}}^{t})}{n}=\frac{\sum_{j=1}^{n}\tau_{j}}{n}$$

where $d(Z_{i}^{t})$ denotes the difference between the current i-th particle $Z_{i}^{t}$ of the t-th iteration and the global optimal solution ${\mathrm{gBest}}^{t}$ of the t-th iteration and $\tau_{j}$ is a statistical factor; a value of 1 for $\tau_{j}$ indicates that $Z_{i}^{t}$ has the same resource configuration strategy as ${\mathrm{gBest}}^{t}$ mapped on the j-th gene, while the converse is the case when $\tau_{j}$ has a value of 0. In this way, the search capability of the algorithm can be adaptively adjusted based on the difference between the current particle and the global optimal particle.

In addition, the cognitive factors of the algorithm $\eta_{pc}^{t+1}$ and $\eta_{gc}^{t+1}$ are set using a linear increase and decrease strategy [33], as ib Equation (14); here, $\eta_{pc}^{star}$ and $\eta_{gc}^{star}$ denote the initial iteration values of the parameters $\eta_{pc}$ and $\eta_{gc}$, while $\eta_{pc}^{end}$ and $\eta_{gc}^{end}$ denote the final values.

**(6) Algorithm Flow**

The detailed process of the APSO-GA is described below.

**Step 1.** Initialize the parameters of the APSO-GA, including the initial population size γ, maximum number of iterations $iters_{max}$, maximum inertia weight $\delta_{max}$, minimum inertia weight $\delta_{min}$, starting and ending values of the acceleration coefficients $\eta_{pc}^{star}$, $\eta_{gc}^{star}$, $\eta_{pc}^{end}$, and $\eta_{gc}^{end}$, and number of positions for the update operation $iNum_{mu}$, $iNum_{pc}$, and $iNum_{gc}$, then randomly generate the initial population.

**Step 2.** Calculate the fitness values of each particle according to Equations (7)–(9), select the optimal one value each particle, and designate the particle with the best fitness values as the global optimal solution in the current generation.

**Step 3.** Update each particle according to Equation (10) and recalculate the fitness of each new particle.

**Step 4.** Update the personal best for each particle; if there exists a better solution than the original global optimal particle, update it.

**Step 5.** If the stopping condition is satisfied, end the algorithm; otherwise, return to Step 3 and continue.

#### 3.4.2. Adaptability of APSO-GA

To ensure adaptability in light of potential changes in edge platform resources and their pricing, direct execution of APSO-GA based on the new pricing table would result in unnecessary overhead. Considering the three plausible scenarios of changes in platform pricing and the correlation between the previous and new configurations, we have designed Algorithm 2 with the aim of obtaining a more appropriate initial population when the pricing table changes in order to facilitate the algorithm’s swift convergence to an optimal scheme for the new environment. The algorithm is as follows, with $new(e_{i}.cpu)$ ($new(e_{i}.mem)$) and $old(e_{i}.cpu)$ ($old(e_{i}.mem)$) representing the previous and updated prices of the platform CPU (memory), respectively.

Algorithm 2 takes two sets of platform prices (before and after the change) as input, along with the configuration scheme of the application before the price adjustment. The output of Algorithm 2 is an initial population suitable for the updated platform prices.

Lines 1 to 2 of the algorithm record whether the prices have increased or decreased. For example, inCpu is set to True in the event of an increase in CPU price. Line 3 maps the original scheme P to the first particle in the initial population $Z_{1}^{1}$, the inverse of Algorithm 1, and sets the number of particles added to the initial population to start from 2. Lines 4 to 17 of the algorithm deal with the first case of price change, in which the CPU(memory) price rises while the memory(CPU) price falls. Line 5 iterates through each gene in the particle and performs the subsequent steps. Line 6 determines whether the current gene prefers memory(CPU) and is deployed on platform $e_{i}$; if it is, then lines 7 to 11 iterate through each edge platform $e_{j}$ and replace the original platform ($e_{i}$) of this gene with other platforms that offer a cheaper CPU(memory) price while keeping the rest of the genes unchanged and incrementing the index by 1. If the conditions in line 6 are not met, line 12 confirms that the current gene does not prefer memory(CPU) and that its deployed platform is not $e_{i}$. If it is, then line 13 replaces it with $e_{i}$ when the platform’s price is higher. Lines 18 to 27 deal with the second case, which occurs when at least one of the prices is elevated. Line 18 iterates through each gene in the particle, and performs the subsequent steps. If the current gene chooses the platform $e_{i}$ and a cheaper platform exists, line 23 replaces $e_{i}$ with the new platform. Lines 28 to 34 deal with the third case, which arises when at least one of the prices is reduced. Line 29 traverses each gene in the particle, and line 31 replaces the platform of the gene with $e_{i}$ if the platform chosen for the current gene is not $e_{i}$ and has a higher price. Lines 35 to 38 determine whether the current population size has reached the threshold. If it has not, the existing particles are used to generate new particles using the APSO-GA crossover operation until the threshold is met. Line 39 returns the generated initial population $Z^{1}$. Then, APSO-GA is executed from Step 2 with this initial population to converge more efficiently towards the new optimal configuration scheme.
**Algorithm 2** Generation of the initial population with adaptive properties.**Input:** $new(e_{i}.cpu)$, $new(e_{i}.mem)$, $old(e_{i}.cpu)$, $old(e_{i}.mem)$, $P=\{p_{1},p_{2},\cdots ,p_{n}\}$.**Output:** $Z^{1}=\left\{Z_{1}^{1},Z_{2}^{1},\cdots ,Z_{\gamma }^{1}\right\}$.1:inCpu←$new(e_{i}.cpu)$ > $old(e_{i}.cpu)$,   inMem←$new(e_{i}.mem)$ > $old(e_{i}.mem)$2:deCpu←$new\left(e_{i}.cpu\right)⩽old\left(e_{i}.cpu\right)$,   deMem←$new\left(e_{i}.mem\right)⩽old\left(e_{i}.mem\right)$3:Map scheme P to particles $Z_{1}^{1}$,   and set index to 24:**if **inCpu(deCpu) **and **deMem(inMem) **then**5:    **for** each $z_{1,k}^{1}\in Z_{1}^{1}$ **do**6:        **if** $z_{1,k}^{1}.cpu$(mem) is more important than $z_{1,k}^{1}.mem$(cpu) **and **$z_{1,k}^{1}.plat==i$ **then**7:           **for** each $e_{j}\in E$ **do**8:               **if** $new(e_{j}.cpu$(mem)) < $new(e_{i}.cpu$(mem)) **then**9:                   $Z_{index}^{1}\leftarrow Z_{1}^{1}$,   $z_{index,k}^{1}.plat\leftarrow j$,   index += 110:               **end if**11:           **end for**12:        **else if** $z_{1,k}^{1}.mem$(cpu) is more important than $z_{1,k}^{1}.cpu$(mem) **and **$z_{1,k}^{1}.plat\neq i$ **then**13:           **if** new( $e_{z_{1,k}^{1}.plat}.mem$(cpu)) > $new(e_{i}.mem$(cpu)) **then**14:               $Z_{index}^{1}\leftarrow Z_{1}^{1}$,   $z_{index,k}^{1}.plat\leftarrow i$,   index += 115:           **end if**16:        **end if**17:    **end for**18:**else if **inCpu** or **inMem** then**19:    **for** each $z_{1,k}^{1}\in Z_{1}^{1}$ **do**20:        **if** $z_{1,k}^{1}.plat==i$ **then**21:           **for** each $e_{j}\in E$ **do**22:               **if** $new(e_{j})$ cheaper than $new(e_{i})$ **then**23:                   $Z_{index}^{1}\leftarrow Z_{1}^{1}$,   $z_{index,k}^{1}.plat\leftarrow j$,   index += 124:               **end if**25:           **end for**26:        **end if**27:    **end for**28:**else if **deCpu** or **deMem** then**29:    **for** each $z_{1,k}^{1}\in Z_{1}^{1}$ **do**30:        **if** $z_{1,k}^{1}.plat\neq i$**and **$new(e_{z_{1,k}^{1}.plat})$ more expensive than $new(e_{i})$ **then**31:           $Z_{index}^{1}\leftarrow Z_{1}^{1}$,   $z_{index,k}^{1}.plat\leftarrow i$,   index += 132:        **end if**33:    **end for**34:**end if**35:**while **index⩽γ **do**36:    $Z_{index}^{1}=Cr\left(Z_{i}^{1},Z_{j}^{1},1\right),\;i,j\in \left(1,index-1\right)$37:    index=index+138:**end while**39:**return **$Z^{1}$

## 4. Experimenal Evaluation

In this section, we evaluate the following questions:

**RQ1:** How accurate is our method in predicting the execution time of functions in serverless applications?

**RQ2:** How efficient is our method in finding the optimal configuration scheme?

**RQ3:** How adaptable is our method in the face of environmental changes?

### 4.1. Experimental Setup

**Platforms.** We built OpenFaas on local servers comprising one master node and five slave nodes. The master node simulates the developers hosting the application to be deployed. This node installs the FireFace, which guides the developers in selecting the optimal configuration settings. The five slave nodes emulate edge platforms providing the services. To account for resource heterogeneity, we assign distinct tendencies of CPU and memory to these nodes. The available sizes of CPU and memory along with their corresponding prices are shown in Table 3. We set the CPU value range to 0.2 for one core in increments of 0.2, while the value range for memory spans from 128 MB to 1024 MB, progressing in 128 MB increments.

**Applications.** The applications used in our evaluation encompass two types, namely, Java applications and DNN-based applications. The Java applications include face detection, license plate recognition, and speech-to-text. The DNN-based applications involve image recognition and utilize three commonly used DNN models: Alexnet, GoogLeNet, and Vgg16. Table 4 presents the serverless functions and SLOs of the applications. In this study, we set reasonable SLOs by considering the execution times of the applications under various configurations.

### 4.2. Accuracy Analysis (RQ1)

In this section, we analyze the accuracy of our prediction model from two aspects. First, we conduct a comparative assessment against other commonly employed regression algorithms to establish the superiority of our method. Second, we evaluate the practical applicability of our trained model by predicting the execution time of functions in serverless applications.

#### 4.2.1. Validation of Superiority

To ascertain the superiority of our prediction model, we employ three widely recognized metrics for evaluating regression algorithms [34]: the Root Mean Square Error (RMSE), coefficient of determination ($R^{2}$), and explained variance score (ExpVar).

RMSE, the average of the differences between predicted and actual values, quantifies the proximity of the model’s predictions to the ground truth. Smaller RMSE values indicate more accurate predictions.

(17) $$\mathrm{RMSE}=\sqrt{\frac{1}{n}\sum_{i=1}^{n}{\left(y_{i}-{\hat{y}}_{i}\right)}^{2}}$$

$R^{2}$ quantifies the goodness of fit. $R^{2}$ yields values within the range of 0 to 1, with values closer to 1 signifying a stronger fit of the model to the data.

(18) $$R^{2}=1-\frac{\sum_{i=1}^{n}{\left(y_{i}-{\hat{y}}_{i}\right)}^{2}}{\sum_{i=1}^{n}{\left(y_{i}-\bar{y}\right)}^{2}}$$

ExpVar is used to evaluate the model’s ability to account for fluctuations in the dataset. A value close to 1 for ExpVar indicates that the model possesses high predictive capability, as it can effectively capture and explain the variations present in the data.

(19) $$\mathrm{ExpVar}=1-\frac{Var{\left(y_{i}-{\hat{y}}_{i}\right)}^{2}}{Var(y)}$$

In Equations (17)–(19), *n* denotes the number of samples, *y* represents the actual value, $\hat{y}$ denotes the predicted value, and $\bar{y}$ represents the mean of the actual value.

We compared our model’s performance against several well-established regression algorithms to validate its superiority:

- Decision Tree (DT): a nonlinear supervised learning algorithm that recursively partitions the data based on features, creating a tree-like structure with decision nodes and assigning labels to samples at the leaf nodes.
- K-Nearest Neighbors (KNN): a nonparametric algorithm that predicts new data points by considering the majority category or means of the k nearest datapoints in the feature space.
- AdaBoost (AD): an ensemble learning method that iteratively combines multiple weak classifiers while assigning higher weights to misclassified samples to progressively build a robust classifier.
- Support Vector Machine with Radial Basis Function Kernel (RBF_SVM): a powerful supervised learning algorithm for regression tasks that employs kernel functions to map data into higher-dimensional spaces and identify an optimal hyperplane for continuous value prediction.

For each algorithm, we employed the dataset constructed in Section 3.3.2 for training, with an 80% split for training and the remaining 20% for evaluation. The experimental results are presented in Table 5.

Table 5 presents the performance evaluation of the regression algorithms across various types of functions. Remarkably, our proposed model (RFR) outperforms all other approaches, as evidenced by the values of $R^{2}$ and ExpVar, which exceed 0.98. This indicates that our model possesses exceptional predictive and explanatory capabilities. The RMSE is contingent on the execution time of different function types. For instance, functions such as Norm, Relu, and Pool exhibit relatively short execution times, typically a few hundred milliseconds, resulting in small RMSE values in the single digits for their corresponding prediction models. However, other function types typically involve longer execution times on the order of a few seconds, leading to relatively larger RMSE values. DT and KNN exhibit commendable prediction performance for certain function types. Conversely, the AD and RBF models are unsuitable for the problem in this paper due to their high prediction errors.

**Figure 6:**
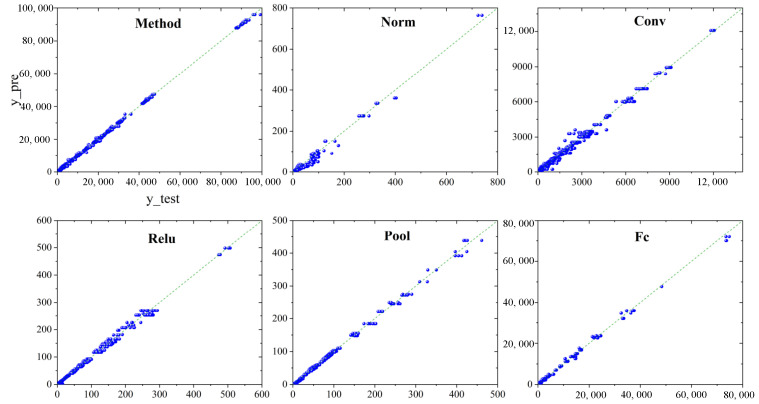
FireFace’s prediction of execution time for different types of functions (Blue circle is the sample, and the green line is the standard line).

In addition, we present error plots depicting the comparisons between our model’s predictions and the actual values. Figure 6 shows that the prediction errors are consistently small, particularly in the prediction of Java methods. This can be attributed to our model’s ability to extract five internal features that effectively represent the complexity of the function. It can be noticed from the figure that data are scarce in the middle segment. This occurrence is linked to instances in which the memory configured to the serverless function is relatively small, for instance, 128 MB, rendering it inadequate to fulfill the function’s basic usage requirements. Consequently, the function is compelled to frequently read and write from the Swap, incurring considerable latency. This observation underscores the critical importance of appropriately allocating resources for serverless functions. In this way, functions can attain short execution time at minimal cost. In contrast, inadequate resource allocation for functions results in inability to meet the SLO, while excessive resource allocation leads to unnecessary overhead.

#### 4.2.2. Validation of Utility

We further validated the utility of our approach by employing a well-trained prediction model to estimate the execution time of each function within the applications used in our experiments. We employed the Absolute Percentage Error (APE) as a metric to measure the accuracy of these predictions. Because the results for all applications would be overly redundant, we present the results for the face detection application here as an illustrative example.

**Figure 7:**
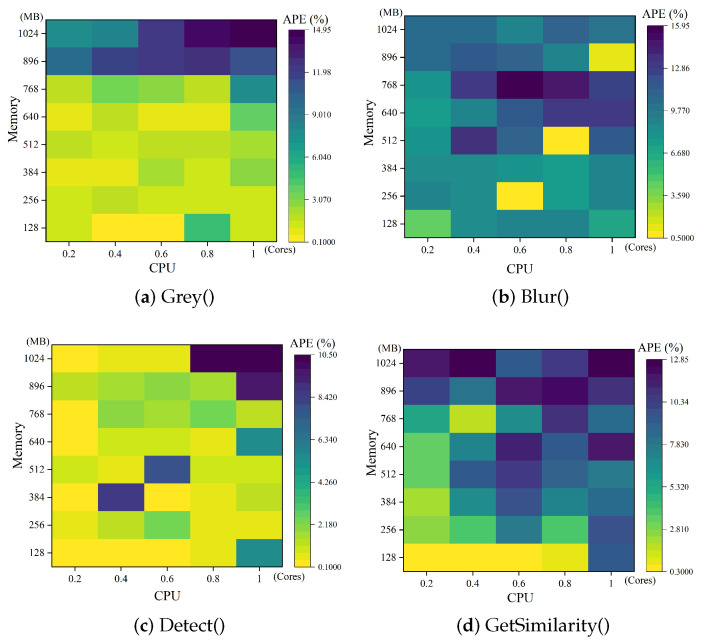
FireFace’s prediction of the execution time of functions in the face recognition application.

Each grid in Figure 7 indicates the difference between the predicted time and actual time under the corresponding CPU and memory, with the darker colors indicating the larger differences. The calculated APE values for these functions are 4.359%, 9.513%, 2.22%, and 7.4974%, respectively. Typically, this level of prediction error results in deviations of only a few tens of milliseconds, which can be considered negligible when compared to overall execution times in the range of thousands of milliseconds. Notably, when the configuration is suboptimal the corresponding APE tends to be lower, typically around 1%. This phenomenon occurs because the scale of the execution time values influences the calculation of APE. In cases where the execution time is higher, the APE tends to be smaller for the same magnitude of prediction error.

### 4.3. Efficiency Analysis (RQ2)

In the preceding section, we focused on analyzing the prediction accuracy of our model. Now, we shift our focus to evaluating the effectiveness of the resource configuration scheme generated by the APSO-GA algorithm. Specifically, we aim to assess whether the scheme can minimize cost while meeting the SLO.

To facilitate the evaluation, we introduce three comparison algorithms for performance comparison:

- Traditional Differential Evolution (DE) [35]: as the discrete problem solved in this paper cannot be directly solved using DE, we convert it into a continuous problem by employing a remainder-based transformation and an adaptive parameter tuning strategy.
- Genetic Algorithm (GA) [36]: this algorithm adopts a binary problem encoding approach, with the number of dimensions corresponding to the available configuration types.
- Particle Swarm Optimization (PSO) [37]: Similar to DE, PSO faces challenges in solving discrete problems; to address this, we apply PSO to the problem by taking the residuals.

All the algorithms share a common fitness function with APSO-GA. The initial population and number of iterations for each algorithm are the same as those of APSO-GA, set at 50 and 300, respectively. The parameters of APSO-GA were initialized with the following values based on previous research [38]: γ = 100, $iters_{max}$ = 300, $\delta_{max}$ = 0.8, $\delta_{min}$ = 0.2, $\eta_{pc}^{star}$=0.9, $\eta_{gc}^{star}$=0.4, $\eta_{pc}^{end}$=0.2, $\eta_{gc}^{end}$=0.9, $iNum_{mu}=6$, $iNum_{pc}=2$, and $iNum_{gc}=1$. Each algorithm is executed twenty times and the iterations and final schemes are recorded. To make the comparison fair, we normalize the results of these algorithms. Due to the vast number of feasible solutions, in this study we consider the optimal scheme from a random set of 100,000,000 configurations as the optimal solution for normalization purposes.

**Figure 8:**
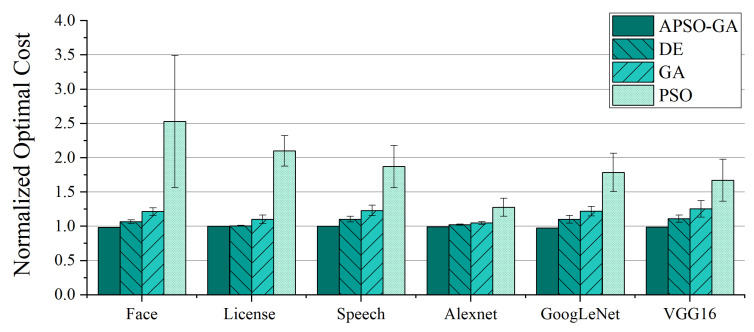
Costs of the resource configuration schemes found by different approaches after 300 iterations; the results are normalized by the cost of the scheme randomly generated 100,000,000 times.

Under the same number of iterations, APSO-GA demonstrates its superiority in discovering better and more stable resource configuration schemes compared to the other algorithms. Figure 8 illustrates the normalized optimal cost of applications for the schemes proposed by each algorithm. Remarkably, our method achieves a reduction 0.008473% to 2.58635% in cost compared to the optimal solution. This could indicate that the scheme found by our algorithm is the truly optimal one. In the case of the License application, the randomized algorithm and our method converge to the same configuration scheme. This can be attributed to this application containing the fewest serverless functions compared to the others, suggesting that in scenarios with fewer serverless functions it may be feasible to find the optimal solution through extensive randomization. These results demonstrate that our method’s performance excels in scenarios which involve additional functions that need to be configured.

Compared to DE, GA, and PSO, APSO-GA reduces the cost of functions by an average of 7.2%, 15.6%, and 44.8%, respectively. While DE and GA can find resource configuration schemes close to the optimal solution for certain applications, they suffer from instability. In contrast, APSO-GA consistently converges to the optimum thanks to its adaptive adjustment of search preferences, which facilitates global evolution during iterations. This underscores the importance of adaptive tuning for heuristic algorithms in achieving better performance. Furthermore, we compared the feasibility rate of the algorithms to determine whether the proposed schemes can satisfy the SLO. PSO exhibits the poorest results, with a feasible solution rate of only 15% on VGG16, indicating that it has difficulty finding feasible solutions under strict SLO conditions. In contrast, our method finds the optimal scheme for every application, illustrating the significant performance improvement achieved by incorporating GA into PSO.

**Figure 9:**
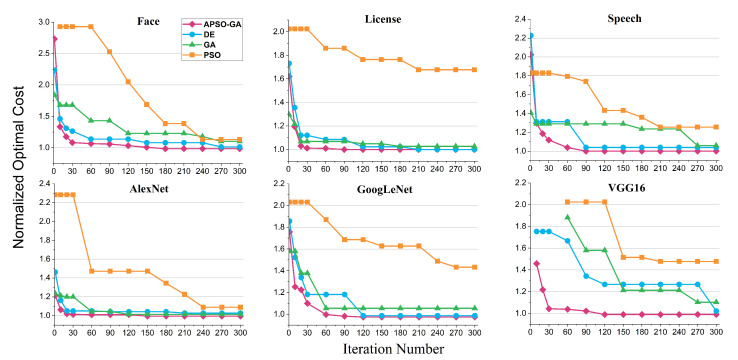
The convergence process of each algorithm when obtaining the best solution in twenty runs.

To compare the convergence speed across different algorithms, the convergence process of each algorithm is displayed when obtaining the best solution in twenty runs. As shown in Figure 9, APSO-GA demonstrates a rapid convergence rate; it usually achieves a feasible scheme after ten iterations, converges to a favorable solution within 90 iterations, and obtains the optimal scheme in approximately 180 iterations. In comparison, DE and GA exhibit a similar convergence rate, typically requiring around 150 iterations to find a better solution. For applications with stringent SLO requirements, such as VGG16, they may require close to 300 iterations to converge. Conversely, PSO exhibits the slowest convergence owing to its inherent difficulties with finding an improved resource configuration scheme. Moreover, it takes approximately sixty iterations for PSO to identify a feasible scheme with tight delay constraints.

We further investigated the influence of SLOs on function execution cost. The variations of SLOs for each application are shown in Table 6, where the tightness of SLOs follows the order SLOs1 > SLOs2 > SLOs3 > SLOs4, where SLOs3 corresponds to our original SLOs (matching the SLOs outlined in Table 4). We used APSO-GA to generate configuration schemes for each application across different SLOs. As in the preceding experiments, the algorithms were all executed twenty times. To facilitate a more intuitive depiction of the effects of SLO adjustments on cost, we normalized the results using the cost of the configuration scheme under SLOs3, i.e., the original SLOs.

**Figure 10:**
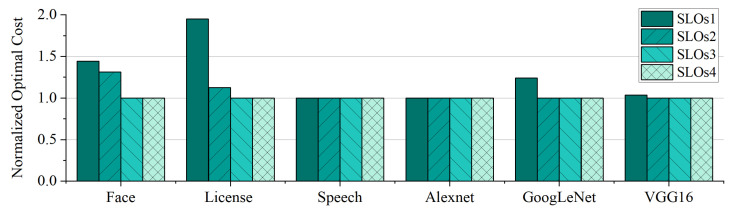
The cost of resource configuration schemes found by APSO-GA for each application across varying SLOs. The results are normalized by the cost of the scheme under SLOs3.

The experimental results are shown in Figure 10; it can be seen that tightening the SLOs leads to cost increases ranging from 3.4% to 95%. This phenomenon arises due to the more stringent SLOs compelling developers to opt for pricier configuration schemes. Contrary to intuition, when SLOs are loose developers tend to favour the original configuration schemes. This tendency is due to the cost being influenced by two key factors: the pricing of the configuration scheme, and the execution time of functions. Opting for a lower-cost configuration could result in insufficient resources, leading to a significant increase in execution time due to substantial read/write operations to and from the swap during serverless function execution. Consequently, even though the per-millisecond price of the chosen configuration scheme may decrease, the cumulative cost is higher due to the substantial increase in execution time.

### 4.4. Adaptive Analysis (RQ3)

In the previous section, we established that APSO-GA can effectively find the optimal configuration scheme for each application with a reasonable number of iterations. In this section, we assess the adaptability of our method by varying the resource prices, as shown in Table 7. Specifically, we consider three scenarios:

- Increasing and Decreasing Prices: we observe how our algorithm adapts to changing prices by increasing and decreasing the price of Platform 5; as indicated in the experimental results in Section 4.3, many functions tend to be deployed on this platform.
- Only Increasing Prices: we solely increase the price of Platform 5 in order to examine whether our algorithm updates the configuration scheme based on the modified platform.
- Only Decreasing Prices: we solely reduce the price of Platform 2, which is the platform with the least number of function deployments.

To demonstrate the enhanced adaptivity of our algorithm through the introduction of Algorithm 2, we compared APSO-GA with and without the use of Algorithm 2; for differentiation, we designate the version of APSO-GA without Algorithm 2 as WAPSO-GA. Both algorithms were configured in the same manner described in the previous section and used the same optimal scheme obtained from the randomized set of 100,000,000 configurations as the benchmark.

**Figure 11:**
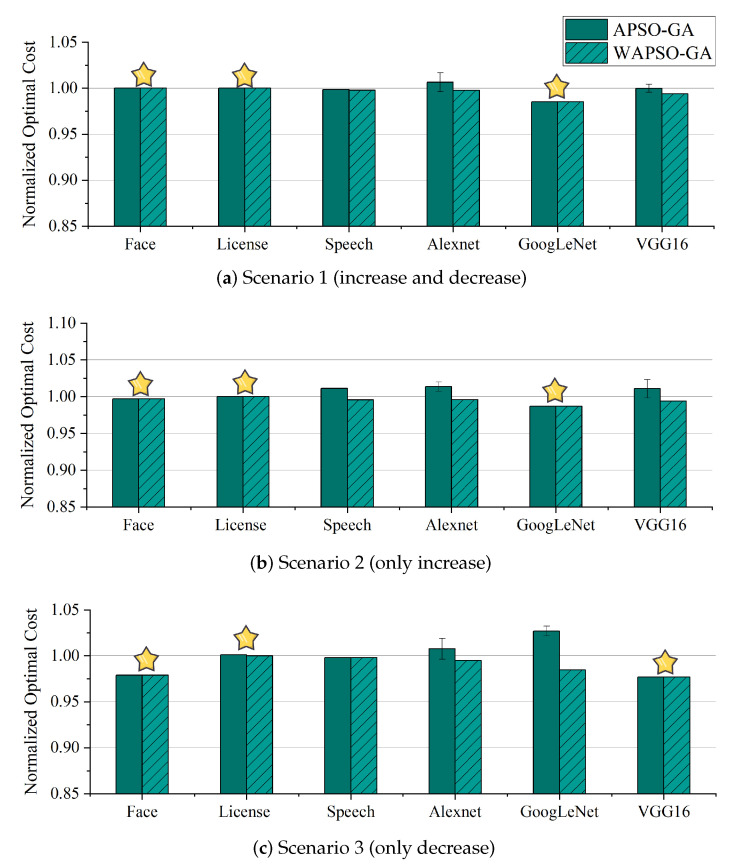
The cost of resource configuration schemes found by APSO-GA and WAPSO-GA under three scenarios after 300 iterations. (Star indicates APSO-GA can achieve the same effect as WAPSO-GA in this scenario.).

Figure 11 shows the normalized optimal costs corresponding to the schemes generated by our algorithm and the WPSO-GA algorithm for different applications in the three scenarios. It can be seen from the figure that WAPSO-GA is able to converge to the optimal solution stably in every scenario. APSO-GA achieves the same effect as WAPSO-GA in half of the scenarios (marked with a star), and has a high chance of finding the optimal solution in the remaining scenarios; however, the stability of the solution cannot be guaranteed. This is because our method is essentially a heuristic algorithm, and as such cannot entirely eliminate the problem of local optimality. However, our algorithm can break through localization through mutation operations, meaning that it has a chance to find the optimal solution, especially with are sufficient iterations. Moreover, the performance difference of APSO-GA compared to WPSO-GA is only 0.086∼10.252% in the scenario when the optimal solution cannot be found. As the purpose of introducing Algorithm 2 was to obtain the most suitable configuration scheme faster, we compared the convergence times.

**Figure 12:**
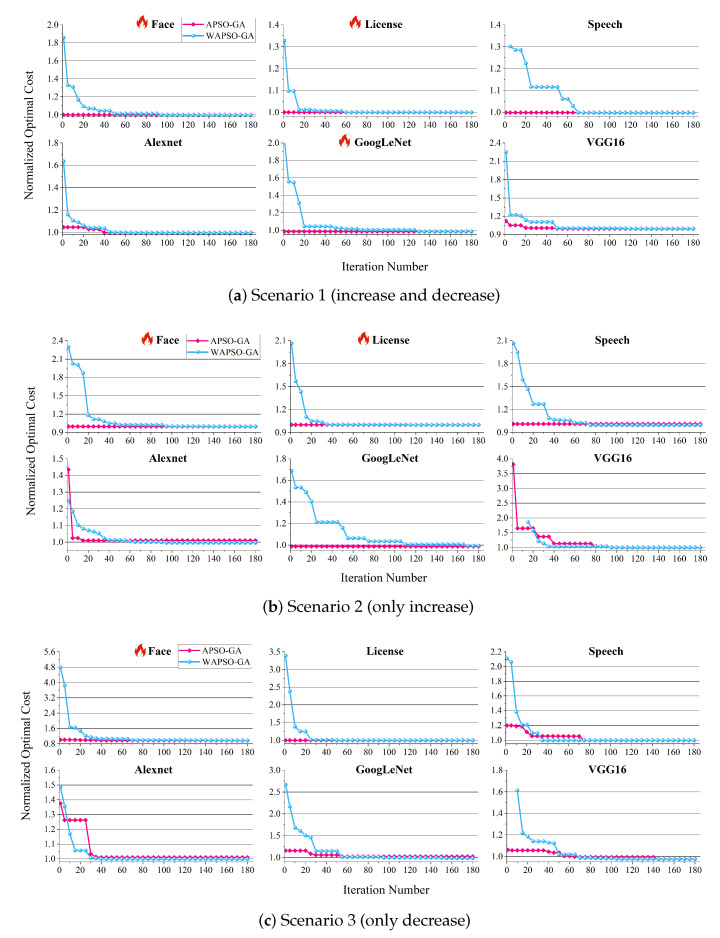
The convergence process of APSO-GA and WAPSO-GA in three scenarios, showing iterations 1 to 180.

As shown in Figure 12, our method can promptly find feasible solutions at the beginning. This can be attributed to the fact that a change in the resource price of the platform does not directly affect the execution time, ensuring that the SLO is satisfied under the original resource configuration scheme. In six out of eighteen scenarios (marked with a flame), the optimal scheme can be directly obtained from the initial population generated by our Algorithm 2. Similar to the findings in the Section 4.3, WAPSO-GA requires 90 iterations to approach a closer-to-optimal scheme, while it takes 180 generations to find the optimal solution. In contrast, our method converges to a scheme close to the optimal solution in approximately 30 iterations.

Overall, the introduction of Algorithm 2 effectively reduces the number of iterations, enabling APSO-GA to quickly adapt to changes in the environment. Moreover, the optimal scheme can be found in most scenarios. Depending on the specific requirements of the scenario, users can decide whether to utilize the initial population generated by Algorithm 2 for faster iterations or opt for random generation to ensure discovery of the optimal solution. This flexibility allows users to tailor the approach based on their needs and constraints.

## 5. Conclusions

The heterogeneity and resource constraints of edge serverless platforms present significant challenges in rationalizing resource configuration for deployment of applications on these platforms. To address this issue, in this paper we have designed and implemented FireFace, a novel framework comprising a prediction module and a decision module. The prediction module utilizes the internal feature information of different function types to predict their execution time under specific configurations. Based on a well-trained prediction model, the decision module then offers optimal resource configuration schemes without requiring real-time execution, thereby minimizing unnecessary overhead. In our experimental evaluations, FireFace consistently outperformed other regression algorithms across all performance metrics, delivering accurate predictions for real-world serverless applications. FireFace generated superior schemes and exhibited the fastest convergence speed compared to other classical algorithms while demonstrating adaptability by quickly responding to changes in the edge environment.

## Figures and Tables

**Table 1:** Information on internal features that affect the execution time of a function.

Application Type	Function Type	Features
Java	Method	blockDepth, percentBranchStatements, complexity, statement, calls
DNN	Normalization (Norm)	input data size, num-features
	Convolution (Conv)	input data size, in-channels, out-channels, kernel-size, stride, padding
	Relu	input data size
	Pooling (Pool)	input data size, output data size
	Fully-Connected (Fc)	input data size, output data size

**Table 2:** Model parameters.

Type	max_depth	max_features	min_leaf	min_split	n_estimators
Method	None	sqrt	2	5	50
Norm	10	log2	1	10	50
Conv	None	sqrt	4	2	50
Relu	None	log2	1	5	50
Pool	None	log2	4	10	50
Fc	10	sqrt	4	10	50

**Table 3:** Resource sizes and prices for edge platforms.

	Platform	1	2	3	4	5
Resource						
Cpu	available size	8 cores	6 cores	4 cores	3 cores	2 cores
	price (core/ms)	0.02	0.04	0.08	0.2	0.4
Memory	available size	4 GB	6 GB	8 GB	12 GB	16 GB
	price (MB/ms)	0.012	0.001	0.0007	0.0004	0.0002

**Table 4:** Serverless functions and SLOs of the applications.

Types	Application	Function	SLO (ms)
	Face detection	Grey(), Blur(), Detect(), GetSimilarity()	30,000
Java	License plate recognition	ColorKmeans(), Oritenation(), Math()	5000
	Speech-to-text	PreProcess(), Recognize(),Compute(), AcceptWave()	20,000
	Alexnet	Module 1 (1∼5 layers), Module 2 (6∼10 layers), Module 3 (11∼15 layers), Module 4 (15∼21 layers)	100
DNN	GoogLeNet	Module 1 (1∼35 layers), Module 2 (36∼87 layers), Module 3 (88∼114 layers), Module 4 (115∼131 layers)	300
	VGG16	Module 1 (1∼20 layers), Module 2 (21∼30 layers), Module 3 (31∼44 layers), Module 4 (45∼51 layers)	600

**Table 5:** Metrics for regression algorithms with different types of functions.

Type	Metrics	RFR	DT	KNN	AD	RBF
Method	RMSE	105.522	1435.09	1112.48	3768.57	2417.49
	$R^{2}$	0.99987	0.97557	0.97984	0.83155	0.91685
	ExpVar	0.99987	0.97557	0.97980	0.83187	0.91703
Norm	RMSE	4.98718	5.23281	5.44878	10.3307	8.92581
	$R^{2}$	0.98014	0.97813	0.97620	0.91478	0.93638
	ExpVar	0.98039	0.97837	0.97660	0.91479	0.93755
Conv	RMSE	63.1270	146.443	97.9260	387.001	318.399
	$R^{2}$	0.99735	0.96568	0.98261	0.79494	0.83778
	ExpVar	0.99735	0.96568	0.98262	0.80520	0.84020
Relu	RMSE	1.88800	8.59310	6.45987	13.6680	10.9591
	$R^{2}$	0.99459	0.85680	0.91183	0.66631	0.81775
	ExpVar	0.99485	0.86694	0.91205	0.67189	0.81940
Pool	RMSE	2.61473	8.07451	5.95543	23.0586	20.2687
	$R^{2}$	0.99620	0.80422	0.98029	0.63808	0.77178
	ExpVar	0.99720	0.80562	0.98092	0.63886	0.77530
Fc	RMSE	382.516	856.530	1060.27	1973.21	3153.00
	$R^{2}$	0.99735	0.98671	0.96709	0.92947	0.71903
	ExpVar	0.99735	0.98671	0.96710	0.92974	0.73916

**Table 6:** SLOs of the serverless applications at different levels of tightness.

	App	Face	License	Speech	Alexnet	GoogLeNet	VGG16
SLOs (ms)							
1 (tightest)		25,000	3600	14,000	10	80	480
ine 2 (tight)		26,000	3700	15,000	80	100	500
ine 3 (original )		30,000	5000	20,000	100	300	600
ine 4 (loose)		40,000	8000	30,000	200	500	1000

**Table 7:** The three scenarios used to investigate edge serverless platform price changes.

Scenario	Platform	Price	
		CPU	Memory
1 (Increases and decreases)	5	0.4 → 0.05 ↓	0.0002 → 0.0009 ↑
2 (Only increases)	5		0.002 → 0.009 ↑
3 (Only decreases)	2		0.001 → 0.0005 ↓

## Data Availability

Data in this paper are available from the corresponding authors upon request.
